# Detection of an undescended parathyroid adenoma with ^18^F-fluorocholine PET/CT

**DOI:** 10.1186/s41824-022-00131-8

**Published:** 2022-05-17

**Authors:** J. Booij, E. W. P. Nijhuis, K. H. ’t Hof

**Affiliations:** 1 Department of Radiology and Nuclear Medicine, University of Amsterdam, Amsterdam UMC, Location Academic Medical Center, Amsterdam, The Netherlands; 2 Department of Pathology, Onze Lieve Vrouwe Gasthuis, Amsterdam, The Netherlands; 3 Department of Surgery, Flevoziekenhuis, Almere, The Netherlands

**Keywords:** Parathyroid adenoma, Undescended, Primary hyperparathyroidism, [^18^F]fluorocholine PET/CT

## Abstract

Surgical excision of a parathyroid adenoma (PTA) is the only curative treatment for primary hyperparathyroidism (PHP). The transition from routine bilateral neck exploration to minimally invasive parathyroidectomy has been made possible by preoperative location techniques, including molecular imaging. Here, we present a case of a 76-year-old man with PHP who underwent a [^18^F]fluorocholine PET/CT scan, which showed a rare undescended PTA at the level of the right carotid bifurcation. After a successful minimally invasive parathyroidectomy, a PTA was confirmed, and the parathyroid hormone level normalized within 24 h. We conclude that it is relevant to locate preoperatively a PTA accurately to assist the surgeon to perform a successful minimally invasive parathyroidectomy.

Surgical excision of a parathyroid adenoma (PTA) is the only curative treatment for primary hyperparathyroidism (PHP). The transition from routine bilateral neck exploration to, a more patient-friendly, minimally invasive parathyroidectomy has been made possible by preoperative location techniques, including molecular imaging (Alvarado et al. 2010; Bioletto et al. 2021). Indeed, a recent meta-analysis showed that preoperatively a PTA can be localized with high sensitivity using ^18^F-fluorocholine PET (Bioletto et al. 2021). Typically, the vast majority of PTAs are located along the thyroid (Petranović Ovčariček et al. 2021). However, undescended PTAs, which are located at or above the carotid bifurcation (Fraker et al. 1990), are rare (< 1% of cases); (Fraker et al. 1990; Lee et al. 2015). Particularly, in such atypical cases, molecular imaging may be relevant to assist the surgeon since this location is unexpected.

A 75-year-old man was diagnosed with a cecum carcinoma, without metastasis, for which he underwent surgery. At follow-up, at the age of 76 years*,* hypercalciemia (serum calcium 3.03 mmol/l) was observed. His clinical workup was compatible with PHP (parathyroid hormone 12.2 mol/l) without clinical signs of PHP. The indication for parathyroidectomy was the severe hypercalciemia. As part of his preoperative workup he initially underwent first-line imaging, existing of sonography of the neck as well dual-tracer [^99m^Tc]tetrofosmin/[^123^I]NaI SPECT/low-dose CT subtraction imaging (Hindié et al. 2021). A PTA was not detected on sonography, and the result of the SPECT examination was inconclusive. Consequently, second-line imaging was performed by acquiring a [^18^F]fluorocholine PET/high-dose CT scan (Hindié et al. 2021) (Fig. 1). This preoperative imaging algorithm was chosen, based on recent recommendations (Hindié et al. 2021). The PET scan was acquired on a Siemens Biograph mCT system, according to recent guidelines (Petranović Ovčariček et al. 2021). The PET/CT scan (upper panel: coronal, sagittal and transaxial PET images; middle and lower panels, CT and overlay of PET/CT images at the same level, respectively) showed an undescended PTA at the level of the carotid bifurcation (right side). After successful excision, PTA was confirmed by histopathological examination (Fig. 2), and the serum calcium and parathyroid hormone levels normalized (2.42 mmol/l and 0.8 pmol/l, respectively) within 24 h.

**Fig. 1:**
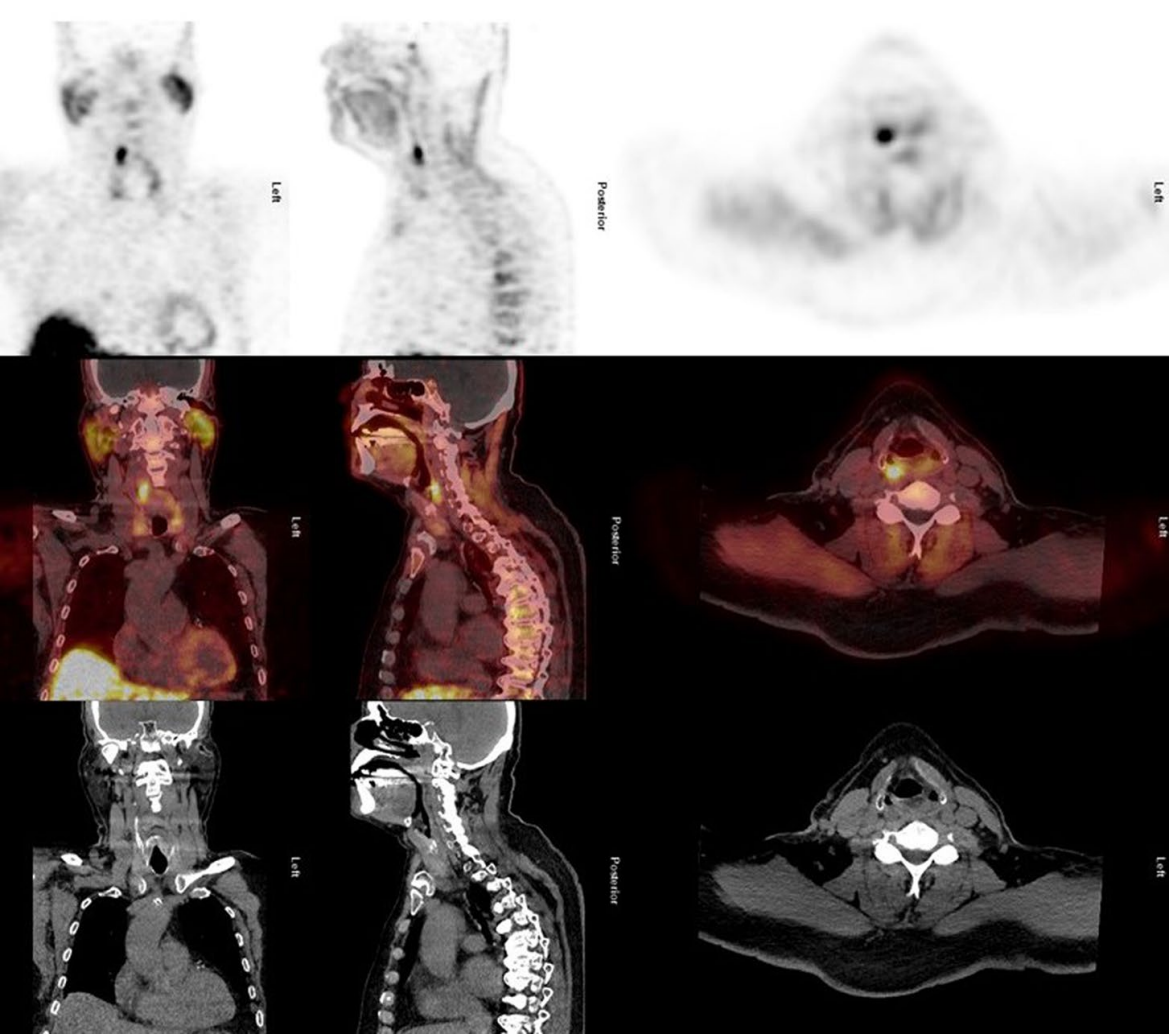
Upper panel: [^18^F]fluorocholine PET images at the level of the undescended PTA. Middle panel: overlay of the [^18^F]fluorocholine PET and high-dose CT images at the level of the undescended PTA. Lower panel: high-dose CT images at the level of the undescended PTA. Left, middle and right columns: coronal, sagittal and transversal views, respectively

**Fig. 2:**
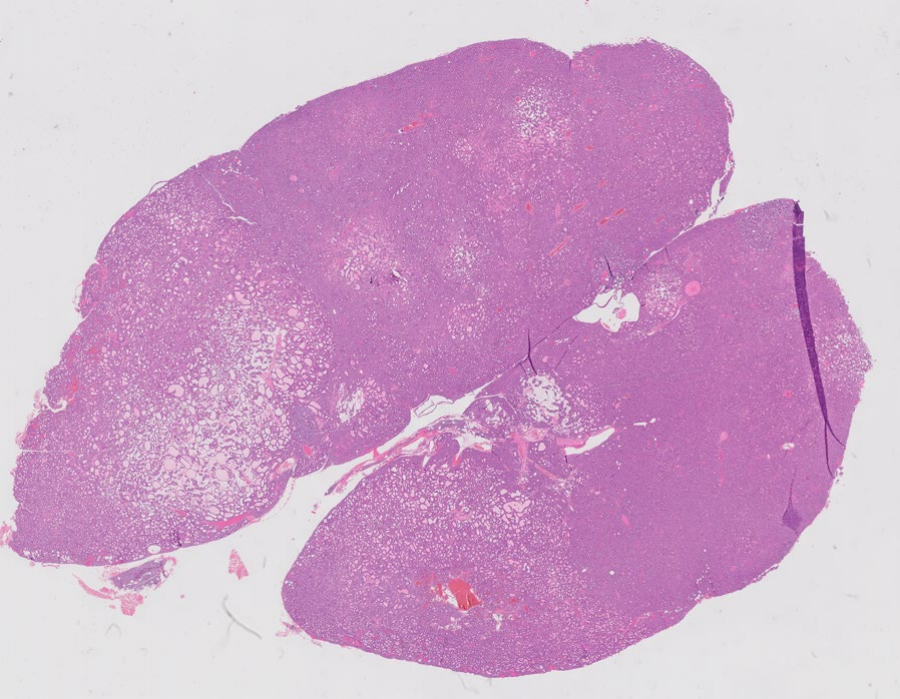
Hematoxylin and eosine staining (magnification 12.5) showing parathyroid tissue (partly solid, partly follicular growth pattern) consistent with a PTA (diameter 2.4 cm)

We conclude that it is relevant to locate preoperatively a PTA accurately to assist the surgeon to perform a successful minimally invasive parathyroidectomy.

## Data Availability

The data supporting the conclusions of this article is included within the article.
